# Research on Axle Type Recognition Technology for Under-Vehicle Panorama Images Based on Enhanced ORB and YOLOv11

**DOI:** 10.3390/s25196211

**Published:** 2025-10-07

**Authors:** Xiaofan Feng, Lu Peng, Yu Tang, Chang Liu, Huazhen An

**Affiliations:** 1 Research Institute of Highway Ministry of Transport, 8 Xitucheng Road, Beijing 100088, China; xf.feng@rioh.cn (X.F.); hz.an@rioh.cn (H.A.); 2 National Center of Metrization for Equipments of Roads and Bridges, Road Traffic Testing Ground of the Ministry of Transport, Jiude Road, Dadu Shexiang, Beijing 101103, China; 3 Automotive Transportation Research Center, 8 Xitucheng Road, Beijing 100088, China; 4 Key Laboratory of MOT of Operation Safety Technology on Transport Vehicles, 8 Xitucheng Road, Beijing 100088, China

**Keywords:** real-time recognition of automotive axle type features, ORB algorithm, FeatureBooster, YOLO

## Abstract

**Highlights:**

**What are the main findings?**

- **Innovation of portable area array acquisition equipment:** Independently developed a comprehensive panoramic image collection and identification system for vehicle chassis, which directly obtains panoramic images of the underside of vehicles through a lateral area array camera and mirror structure. The system can be deployed in 20 min without road embedding.
- **ORB feature matching + FeatureBooster feature enhancement + YOLOv11n image detection scheme achieves breakthrough accuracy:** In the identification of vehicle axle types, the model achieved a precision of 0.98, a recall of 0.99, and an mAP@50 of 0.989 ± 0.010, demonstrating superior performance compared to traditional methods.

**What is the implication of the main finding?**

- **Addressing toll dispute pain points:** Accurately distinguishing between drive axles and driven axles, providing reliable visual evidence for toll booth entrances that charge based on axle types.
- **Empowering real-time traffic management:** The processing result for a single vehicle can be output within 1.5 s after the vehicle passes (with 99% accuracy), which can help toll booths quickly identify vehicle types and confirm charging standards on-site.

**Abstract:**

With the strict requirements of national policies on truck dimensions, axle loads, and weight limits, along with the implementation of tolls based on vehicle types, rapid and accurate identification of vehicle axle types has become essential for toll station management. To address the limitations of existing methods in distinguishing between drive and driven axles, complex equipment setup, and image evidence retention, this article proposes a panoramic image detection technology for vehicle chassis based on enhanced ORB and YOLOv11. A portable vehicle chassis image acquisition system, based on area array cameras, was developed for rapid on-site deployment within 20 min, eliminating the requirement for embedded installation. The FeatureBooster (FB) module was employed to optimize the ORB algorithm’s feature matching, and combined with keyframe technology to achieve high-quality panoramic image stitching. After fine-tuning the FB model on a domain-specific area scan dataset, the number of feature matches increased to 151 ± 18, substantially outperforming both the pre-trained FB model and the baseline ORB. Experimental results on axle type recognition using the YOLOv11 algorithm combined with ORB and FB features demonstrated that the integrated approach achieved superior performance. On the overall test set, the model attained an mAP@50 of 0.989 and an mAP@50:95 of 0.780, along with a precision (P) of 0.98 and a recall (R) of 0.99. In nighttime scenarios, it maintained an mAP@50 of 0.977 and an mAP@50:95 of 0.743, with precision and recall both consistently at 0.98 and 0.99, respectively. The field verification shows that the real-time and accuracy of the system can provide technical support for the axle type recognition of toll stations.

## 1. Introduction

Highways are the lifeline of economic development. However, with the continuous increase in traffic volume, particularly the growing prevalence of overloaded and excessively loaded vehicles, asphalt pavements commonly experience early damage [1], severely affecting the service life of the road network and driving safety. The key factors leading to structural damage of the pavement are vehicle axle types and axle loads [2]. To more scientifically and fairly reflect the actual wear caused by vehicles on the roads, the Ministry of Transport of the People’s Republic of China (MOT) issued and implemented the industry standard “Vehicle classification of the toll for highway” (JT/T 489-2019) in 2019 [3], adjusting the charging method for vehicles to a uniform fee based on axle type. This classification is primarily based on the national standard “Limits of dimensions, axle load and masses for motor vehicles, trailers and combination vehicles” (GB 1589-2016) [4]. In 2021, the MOT further released the “Administration Regulations on Road Transport of Over-dimensional and Overweight Vehicles,” [5] clearly stating that the total weight of trucks is mainly determined by the number of axles, the number of drive axles, and the configuration of single or dual tires. These series of policies underline the urgency of achieving rapid and accurate identification of vehicle axle types at toll booth entrances.

Regarding vehicle toll charges, when the license plate is associated with vehicle information, it is possible to determine vehicle details through license plate recognition [6], thereby establishing the toll standards. However, in China, particularly for center-axle trailer combination, the tractor and the trailer often have different license plates, and the trailer’s license plate is frequently obstructed. Therefore, relying solely on license plate recognition to implement the toll standards is not a viable strategy.

Currently, researchers both domestically and internationally have proposed various automated identification solutions, such as embedding inductive coils [7], infrared detection [8], piezoelectric sensors [9], lidar [10], and image recognition [11]. However, although these methods can identify the number of axles, there are still significant challenges in effectively distinguishing between drive and driven axles. Additionally, most equipment is installed on both sides of the passage, making it susceptible to being obstructed by vehicle coverings; some equipment needs to be embedded in the center of the passage, which not only increases implementation costs and construction complexity but also leads to traffic congestion during installation. These limitations highlight the need for a more practical and efficient approach to vehicle axle recognition.

Furthermore, during industry management and equipment calibration processes, it is necessary to retain clear, intuitive image evidence to address potential disputes that may arise later. Due to their capacity to impartially and accurately document and reconstruct incident scenarios, visual media such as images and videos are frequently employed as pivotal forms of evidence in adjudicating disputes within the transportation sector. To address these issues, this study aims to develop a portable non-embedded device for axle type image recognition. This design avoids the high costs and traffic disruptions associated with embedded installations, while simultaneously enabling the acquisition of panoramic under-vehicle images and axle type features. The core technical challenges to be addressed include: (1) achieving robust under-vehicle panoramic image stitching under a constrained field of view using image stitching techniques and (2) realizing accurate identification of axle type features based on object detection technology.

## 2. Related Work

### 2.1. Image Stitching Algorithms

In practical applications, the proximity of a vehicle’s chassis to the ground often results in a restricted field of view, making it hard to capture a complete image of the chassis in a single acquisition. As a result, image stitching algorithms are required to combine multiple overlapping images into a coherent and comprehensive chassis panorama. A variety of image stitching algorithms have been developed, such as SIFT [12], SURF [13], ORB [14], KAZE [15], and SuperPoint [16]. The ORB (Oriented FAST and Rotated BRIEF) algorithm, in particular, is extensively utilized in image stitching applications owing to its computational efficiency and robust performance [17]. For instance, Zhang et al. [18] proposed a damage reconstruction image stitching technique based on ORB feature extraction and an improved MSAC algorithm, aimed at efficiently assessing the damage to spacecraft caused by micro-meteoroids and orbital debris. Similarly, Luo et al. [19] developed a novel ORB-based image registration framework demonstrating superior accuracy and faster processing for UAV image stitching across diverse environments including urban zones, roadways, structures, farmlands, and forests. Li [20] proposed a fuzzy control-based ORB feature extraction algorithm (OFFC) to mitigate issues related to overly dense and overlapping feature points, enhancing matching accuracy under conditions such as motion blur, illumination variations, and high-texture similarity. Zhao et al. [21] improved PCB image stitching by enhancing ORB feature description with BEBLID descriptors and optimized matching, achieving robust and accurate registration for quality assessment. Chen et al. [22] proposed a real-time video stitching method based on ORB feature detection combined with secondary fusion to reduce ghosting and improve stitching quality while ensuring real-time performance for robotic vision. Mallegowda M et al. [23] compared serial and parallel implementations of an ORB-based image stitching algorithm, showing that parallel processing significantly accelerates runtime for real-time applications in autonomous vehicles. In the context of vehicle axle recognition systems deployed at toll station entrances, rapid and precise acquisition and processing of vehicle images are essential for determining toll criteria accordingly. Therefore, this paper applies an enhanced ORB-based image stitching method to deliver efficient and reliable data support for subsequent vehicle chassis analysis and identification.

### 2.2. Object Detection Algorithms

The mainstream algorithms in the field of image feature recognition include Convolutional Neural Networks (CNN), YOLO (You Only Look Once), R-CNN and its variants (such as Faster R-CNN [24] and Mask R-CNN [25]), SSD [26] (Single Shot MultiBox Detector), and Transformers [27] (such as Vision Transformer). Among these, YOLO was proposed by Joseph Redmon et al. [28] and is widely used due to its efficient real-time processing capabilities, user-friendly framework, and high detection accuracy. Ye et al. [29] designed a novel online detection system based on the YOLO algorithm, which can efficiently achieve real-time monitoring and recognition of automotive components, significantly improving the accuracy of logistics and operational efficiency in the automotive industry. Mo et al. [30] proposed an improved detection model based on YOLO, which demonstrates superior detection capabilities for distant targets, occluded objects, dense pedestrian areas, and multi-vehicle scenarios. Zhao et al. [31] introduced an improved YOLOv8n algorithm that significantly enhances the detection performance of hazardous objects under vehicles, meeting the high accuracy and real-time requirements for under-vehicle safety inspections. Almujally et al. [32] developed a nighttime vehicle tracking system using YOLOv5 with MIRNet enhancement and SIFT-based matching, achieving 92.4% (UAVDT) and 90.4% (VisDrone) detection accuracy. Raza et al. [33] evaluated YOLO-V11 for foggy vehicle detection, achieving 73.1% mAP on DAWN and 47% F1 on FD datasets while balancing speed (26 FPS) and accuracy. Zhang et al. [34] proposed LLD-YOLO for low-light vehicle detection, integrating DarkNet and attention modules, achieving 83.3% mAP on ExDark (4.5% higher than baseline). Pravesh [35] achieved 97.28% accuracy (F1 95.78%) for low-light firearm detection using a YOLOv11 framework with triple-stage enhancement, surpassing baseline models. He et al. [36] applied the YOLOv11 model to high-resolution remote sensing images, demonstrating rapid convergence of loss functions and achieving high precision (0.8861), recall (0.8563), and mAP scores (0.8920 at 50% threshold), indicating strong accuracy and robustness in multiclass object detection. Nguyen et al. [37] introduced UFR-GAN, a lightweight multi-degradation restoration framework integrating transformer-based feature aggregation and frequency-domain contrastive learning, which improved restoration quality and enhanced vehicle detection accuracy by 23% when combined with YOLOv11 under adverse weather. Chen et al. [38] proposed ReDT-Det, a Retinex-guided illumination differential transformer detection network for nighttime UAV vehicle detection, combining image enhancement and feature fusion modules to effectively detect small and medium-scale objects, outperforming state-of-the-art methods on multiple challenging datasets. Given the YOLO algorithm’s advantages of strong real-time performance, high robustness, excellent detection accuracy, and ease of deployment, this paper employs the YOLO algorithm to implement image feature recognition of vehicle chassis.

### 2.3. Research Gap and Contributions

At present, although some researchers have conducted studies on vehicle axle type recognition [39,40,41,42], there is limited research on real-time acquisition of chassis images and axle information at toll station sites. In view of this, the present article aims to propose a panoramic chassis image detection technology. The main contributions of this work are threefold: (1) a portable area-array imaging device is developed for efficient under-vehicle image capture; (2) an enhanced ORB-based stitching pipeline with feature boosting for robust panoramic image generation; and (3) YOLOv11 is integrated for accurate and real-time axle type recognition, validated through extensive on-site experiments. The proposed panoramic chassis image detection technology can provide a basis for toll collection and overload control management on highways, as well as technical support for the future implementation of unmanned and free-flow toll stations.

## 3. Methods

### 3.1. Equipment Development

**Figure 1:**
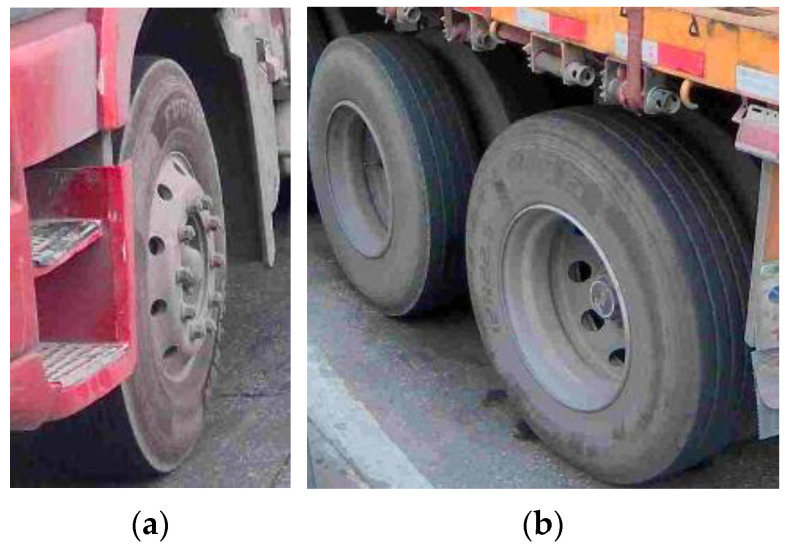
The tire types of goods vehicles. (**a**) Single tire; (**b**) dual tire.

In the management of freight vehicles, the complexity of axle types and weight limit requirements often leads to errors or disputes during the charging process. The MOT has issued standards for the identification of overloaded and overweight freight vehicles on highways [43], along with Table A1 in Appendix A that outlines the corresponding number of drive axles and trailing axles for different vehicle models. The weight limit of a vehicle is primarily determined by the number of trailing axles, drive axles, and the number of tires. It is important to note that there are significant structural differences between single tires and dual tires (see Figure 1); however, since the trailing axles and drive axles are located at the bottom of the vehicle, direct identification is challenging, which poses difficulties for actual inspection and management. Existing solutions, such as inductive loops and piezoelectric sensors embedded in the road, require extensive construction, cause traffic disruption, and provide no visual evidence. In addition, side-mounted vision systems are often obstructed by vehicle attachments. Therefore, this paper aims to develop a system capable of directly identifying a vehicle’s trailing and drive axles using advanced image feature stitching and recognition techniques.

In the process of developing the equipment, several key considerations were taken into account:

- Accurate identification and acquisition of axle type information are essential for axle-based charging. For industry management and equipment performance testing departments, having clear, intuitive, and easily distinguishable evidence is vital to resolving any potential disputes that may arise. Therefore, this article designs an imaging system that captures panoramic vehicle underside images through an image stitching algorithm, providing direct visual evidence for axle-based toll collection. Moreover, it supplies high-quality, realistic data to support subsequent axle feature recognition models in practical application scenarios.
- The installation of embedded axle-type identification devices, a process involving positioning, trenching, embedding, sealing, and backfilling within the lane, typically requires no less than one day to complete. During this period, traffic flow is interrupted, significantly impacting toll station operations. To facilitate rapid deployment and minimize disruption, the equipment proposed in this article is designed to be surface-mounted rather than embedded, achieving operational readiness in under 20 min. This approach substantially reduces both installation complexity and cost by avoiding extensive roadwork. Nevertheless, a key challenge in non-embedded systems is the limited field of view resulting from the low clearance of vehicle chassis. To overcome this limitation, the developed system employs a horizontally oriented camera combined with a reflective mirror, expanding the effective viewing range.
- At toll station entrances, especially in high-traffic scenarios, vehicles often queue in close proximity, which complicates the task of distinguishing individual vehicles by the acquisition device. This proximity increases the risk of multiple adjacent vehicles being misidentified as a single entity. Therefore, a laser vehicle separator is deployed to support vehicle distinction.

**Figure 2:**
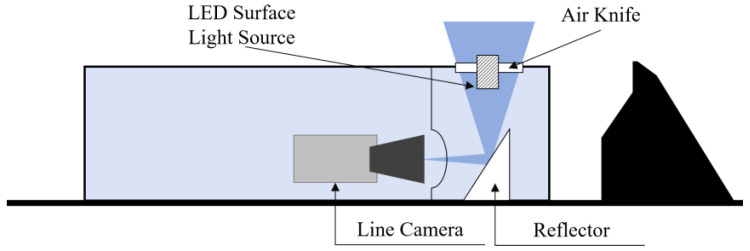
Schematic diagram of vehicle chassis image acquisition equipment.

In summary, this article designs the design and implementation of a panoramic imaging detection device tailored for vehicle underbody inspection. Figure 2 shows a schematic of the image acquisition unit. Figure 2 The portable image capture device mainly comprises a camera, a protective housing, a reflective mirror, and an LED surface-based lighting unit. The core imaging component is a color area-scan CMOS camera equipped with a global shutter. It offers a resolution of 2048 × 160 pixels, a maximum acquisition frequency of 30 Hz, and a signal-to-noise ratio exceeding 39 dB. This camera supports the acquisition of high-resolution, wide-field-view imagery. The horizontal camera orientation, combined with a reflective mirror, extends the effective viewing angle, reducing optical distortion and improving the clarity of captured vehicle chassis images. The integrated LED surface light functions as supplemental illumination, automatically activating under low ambient light conditions. Additionally, the protective cover incorporates impact-resistant sloped surfaces designed to mitigate damage in case of accidental vehicle overrun.

When utilizing the device, its portable design allows for non-embedded deployment by simply placing it on the road surface at the center of the toll station entrance lane. According to the “Administration Regulations on Road Transport of Over-dimensional and Overweight Vehicles” [5], the maximum allowable length for non-overlimit vehicles is 18.1 m. Therefore, the device is placed about 20 m from the barrier gate. This distance ensures most vehicles can be fully imaged and their chassis features extracted before reaching the barrier.

Upon image acquisition, the data are transferred to a host computer for subsequent stitching and object detection. The host computer is equipped with an NVIDIA GeForce RTX 3060 GPU (12 GB VRAM), a multi-core CPU (minimum 10 cores, 20 threads), 32 GB DDR4 RAM, and a 1 TB NVMe SSD. This configuration provides sufficient computational power for processing large image datasets and accelerating complex algorithms.

Model training was performed on a separate workstation with an NVIDIA GeForce RTX 3090 GPU (24 GB VRAM), an Intel Xeon Gold 6226R CPU (2.90 GHz), and 128 GB DDR4 RAM.

### 3.2. Image Processing Algorithm

**Figure 3:**
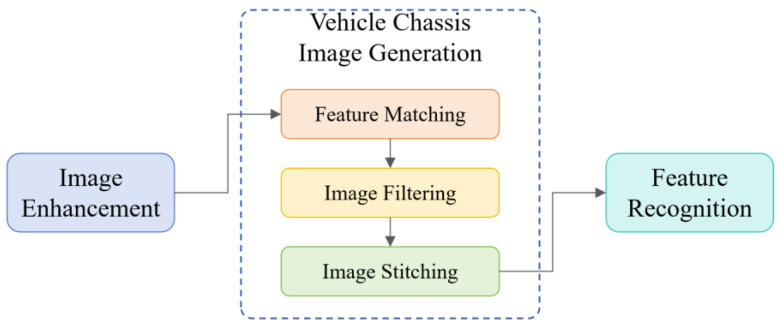
Image Processing Workflow Diagram.

This chapter provides a detailed description of the data processing workflow for images captured by area scan cameras, including image enhancement, vehicle chassis image generation, and feature recognition, as shown in Figure 3. The workflow starts with image enhancement to improve image quality and facilitate subsequent feature extraction. The system then generates the panoramic vehicle chassis image This involves extracting salient features from the enhanced images and employing matching algorithms to identify corresponding feature points across different frames. To reduce computational complexity and improve stitching results, keyframes are selected based on the extracted features, ensuring representative coverage of the overall image content. These keyframes are then fused to create a complete panoramic image of the vehicle chassis. Finally, axle type information is extracted from the stitched result using feature recognition techniques. This workflow enables efficient and accurate detection and identification of vehicle undersides, providing reliable data support for subsequent vehicle classification and management. This chapter presents a systematic solution for image data processing using area scan cameras, ensuring high quality and accuracy in the final results.

#### 3.2.1. Image Feature Enhancement

During the feature extraction process of vehicle chassis images, the following issues may arise:

- Some areas may be too dark or too bright, making it difficult to capture detailed information.
- The presence of salt-and-pepper noise or other random noise in the images can affect the accurate detection of feature points.
- Insufficient overall contrast can lead to key details being blurred, making it challenging to differentiate between various structural features.

To overcome these challenges, this article adopts the following image enhancement techniques:

- Utilizes the Median Filtering algorithm to remove salt-and-pepper noise from the input images while smoothing the images to preserve edge information.
- Employs the CLAHE algorithm (Contrast Limited Adaptive Histogram Equalization) to enhance the contrast of the image in local regions through histogram equalization. This approach reduces the impact of lighting on image quality while avoiding noise amplification. The formula for CLAHE is:

(1) $$H_{CLAHE}(x,y)=\frac{H_{in}(x,y)-H_{min}}{H_{max}-H_{min}}\times 255$$

In the formula, *H_in_*(*x*, *y*) represents the grayscale value of the pixel points within the local area of the input image, while *H_min_* and *H_max_* denote the minimum and maximum grayscale values within the current local region, respectively. *L_max_* and *L_min_* refer to the maximum and minimum grayscale values of that local region. In this article, the size of the local area is set to 8 × 8.

- Utilizes Gamma Correction for non-linear adjustments of image brightness to improve the visibility of details in the dark regions of the chassis images. The formula for gamma correction is:

(2) $$O=I^{\gamma }$$

In the formula, *O* represents the output pixel value, *I* is the normalized pixel value of the image, and γ is the gamma coefficient, which is set to 0.8 in this article.

#### 3.2.2. Vehicle Chassis Image Generation

1. Image Feature Matching

When the vehicle passes through the image acquisition system, many area array images will be generated. Consequently, the feature matching process consumes a significant amount of computational resources. Thus, it is essential to adopt an efficient feature matching algorithm to effectively analyze vehicle chassis imagery. The ORB algorithm is widely used in real-time feature matching due to its computational efficiency and strong performance. Its speed makes it a preferred choice, especially in applications that require quick responses. However, despite its speed, the accuracy of feature matching can be easily affected by interference. Therefore, this paper aims to enhance the accuracy of feature matching by applying an improved ORB algorithm to better meet the needs of practical applications.

**Figure 4:**
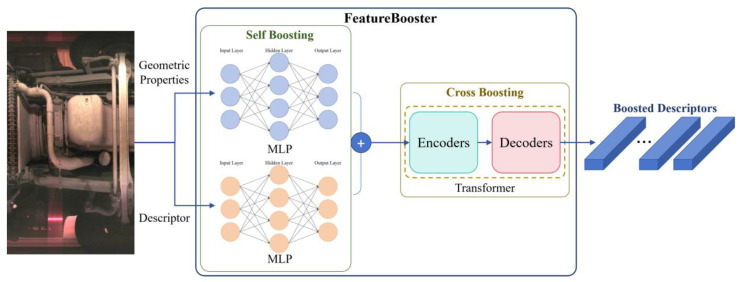
The architecture diagram of the FB algorithm.

In 2023, Wang et al. [44] proposed the FeatureBooster (FB) algorithm. Based on existing feature extraction descriptors, this algorithm employs a lightweight neural network to perform self-enhancement and cross-enhancement on the original keypoints and descriptors, aiming to generate higher-quality feature descriptors. The overall architecture is shown in Figure 4.

In the self-enhancement stage, FB employs two multilayer perceptrons (MLPs) to perform nonlinear transformations on the original feature descriptors and geometric attributes of the image, respectively. These are then combined through addition to generate a self-enhanced descriptor that integrates both visual and geometric information. The forward propagation of the MLPs is expressed as Equation (4):

(3) $$y^{0}=x,y^{\left(l\right)}=\sigma (W^{\left(l\right)}y^{\left(l-1\right)}+b^{\left(l\right)})$$

In the equation, *l* denotes the layer number of the network, *y*^(*l*)^ represents the output of the *l*-th layer, *W*^(*l*)^ is the weight matrix of the *l*-th layer, *b*^(*l*)^ is the bias vector, and *σ* is the activation function, which is the ReLU function in FB.

In the cross-enhancement stage, FB leverages a lightweight Transformer to strengthen the interrelationships, spatial layout, and dependencies among feature points, thereby significantly improving overall feature matching performance. The core idea of the Transformer is to capture global dependencies among elements in a sequence through the multi-head self-attention mechanism. Its architecture mainly consists of an encoder and a decoder, where each encoder layer includes a multi-head self-attention module and a feed-forward neural network, with residual connections and layer normalization employed to ensure training stability. The computation formula for self-attention is as follows:

(4) $$Attention(Q,K,V)=Softmax(\frac{QK^{T}}{d_{k}})$$

In the equation, *Q* denotes the query vector, *K* is the key vector, *V* is the value vector, and *d_k_* is the dimension.

The FB is employed to improve the average precision of feature descriptors while ensuring that the enhanced descriptors consistently outperform their original counterparts. To achieve this objective, the loss function incorporates both Average Precision Loss and Boosting Loss, which are formulated as follows:

(5) $$L=L_{AP}+\lambda L_{Boost}$$

(6) $$L_{AP}=1-\frac{1}{N}\sum_{i=1}^{N}AP(d_{i}^{tr})$$

(7) $$L_{boost}=\frac{1}{N}\sum_{i=1}^{N}\max(0,\frac{AP(d_{i})}{AP(d_{i}^{tr})}-1)$$

where *L* is the loss function of FB, *L_AP_* is the Average Precision Loss, *L_boost_* is the Boosting Loss, λ is a coefficient set to 10 during training. *N* is the number of keypoints, $d_{i}^{tr}$ is the enhanced descriptor of the i-th keypoint produced by the FB, and AP is the calculation of average precision. *d_i_* represents the original input descriptor. This loss term encourages the model to generate more discriminative descriptors, ensuring that correct matches are ranked higher than incorrect ones.

In summary, FB algorithm can augment the ORB by increasing robustness to illumination changes, improving adaptability to viewpoint variations, producing high-dimensional descriptors, and optimizing matching strategies. Consequently, this article adopts the ORB algorithm combined with FB to perform image feature matching.

2. Image Filtering Based on Video Keyframes

When a vehicle passes through a toll station, it may accelerate, decelerate, or come to a complete stop. A decrease in vehicle speed leads to an increase in the overlapping areas between consecutively sampled images. Analyzing all images will not only cause huge computational burden, but also lead to information redundancy. Therefore, keyframe selection strategy [45] is used to alleviate computational overhead while ensuring that critical scenes and important changes are adequately captured. Keyframes at different time points are selected, and the overlap between images is calculated based on a feature matching method, as shown in Equation (3). The current frame is designated as a keyframe when the variation surpasses a predefined threshold.

(8) $$D=Metric(F_{t},F_{t+n})$$

In the equation, *D* represents the overlap degree, *Metric* refers to the function applied for overlap computation, *F_t_* is the feature vector of the image at frame t, and *n* is the sampling interval.

3. Image Stitching

First, the RANSAC algorithm [46] is used to improve the accuracy and robustness of the matching results. Then, a weighted image stitching algorithm based on linear constraints is applied to complete the image fusion. Since images captured by the area-scan camera have strong temporal correlation, sequential fusion can produce a panoramic image of the vehicle chassis. Based on feature points obtained from feature matching, the homography matrix *H* is estimated using the least squares method. Assuming the pixel values in the overlapping region of the images are *I*_1_(*x*, *y*) and *I*_2_(*x*, *y*), the weighted fusion formula is usually expressed as:

(9) $$\begin{array}{l} I_{result}(x,y)=\omega_{1}I_{1}(x,y)+\omega_{2}I_{2}(x,y)\\ \omega_{1}+\omega_{2}=1 \end{array}$$

*I_result_*(*x*, *y*) represents the pixel value in the overlapping region after weighted fusion. *ω*_1_ and *ω*_2_ are the weights of the two images, respectively, and they satisfy the condition *ω*_1_ + *ω*_2_ = 1. The weight functions are designed to create a smooth transition and eliminate visible seams in the overlapping region. The weight for the first image (ω_1_) increases linearly from 0 to 1 across the overlap, while the weight for the second image (ω_2_) decreases linearly from 1 to 0. This ensures that each image’s contribution is dominant near its respective side of the overlap and gradually diminishes. And this results in a natural blend that mitigates discontinuities in illumination and color. The weight function is calculated as follows:

(10) $$\omega_{1}=\frac{x-x_{\min}}{x_{\max}-x_{\min}},\omega_{2}=1-\omega_{1}$$

*x* represents the horizontal coordinate of a pixel, and *x_max_* and *x_min_* are the maximum and minimum horizontal coordinates of the pixels in the overlapping region.

#### 3.2.3. Axle Type Feature Recognition Algorithm Based on YOLOv11

The length and speed of the vehicle affect the stitching dimensions of the chassis image. Additionally, although the underbody image acquisition equipment is equipped with auxiliary lighting, complex and variable on-site shooting environments can affect image quality to varying degrees. Therefore, the designed vehicle underbody image feature recognition algorithm must be robust to multi-scale and deformation variations, and adaptable to varying lighting conditions and image quality.

**Figure 5:**
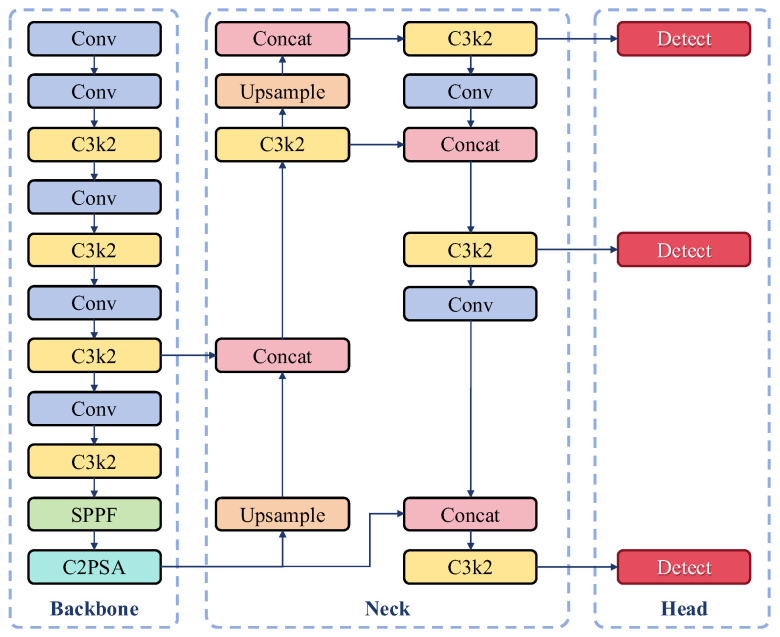
YOLOv11 Algorithm Architecture Diagram.

The YOLO algorithm [28], features an end-to-end single-stage detection framework, delivering outstanding real-time performance and highly efficient object localization capabilities. Through continuous updates and iterations, the YOLOv11 algorithm was released by Ultralytics in 2024. Compared to previous versions, YOLOv11 undergoes comprehensive upgrades in network architecture, training strategies, and inference processes. These enhancements elevate detection performance, strengthen model robustness, and improve adaptability to complex scenarios. The overall architecture of the algorithm is illustrated in Figure 5.

YOLOv11 continues the classic Backbone-Neck-Head three-stage architecture design of the YOLO series, introducing a series of innovative optimizations across its modules that lead to breakthroughs in both accuracy and efficiency.

The Backbone employs the improved C3K2 module. This module utilizes a dual-branch design—where a 3 × 3 convolution captures local features and a 1 × 1 convolution enables channel interaction—to facilitate multi-scale feature fusion. Additionally, the new Cross-Level Pyramid Slice Attention (C2PSA) module enhances global feature modeling capabilities through a multi-head attention mechanism, thereby enhancing model performance in complex scenarios and for occluded objects. Furthermore, the SPPF module reduces computational load while maintaining the receptive field by employing cascaded small pooling kernels (5 × 5).

The Neck section adopts an adaptive feature pyramid structure, which optimizes the training process via a dynamic weight allocation mechanism. This mechanism prioritizes localization accuracy in the early training stages and shifts focus to classification accuracy in the later stages, thus accelerating convergence. Lightweight attention modules are also embedded to strengthen the feature response for small objects.

In the Head module, the classification and regression branches are designed using a fully decoupled depthwise separable approach. This approach decomposes large-kernel convolutions into multiple parallel branches to circumvent the high computational cost of their direct use. By integrating a dynamic kernel selection mechanism with the optimized Distribution Focal Loss (DFL), the detection performance is significantly enhanced.

These collective enhancements render YOLOv11 exceptionally well-suited for tackling the challenges of multi-scale object detection in complex environments, a common scenario in vehicle chassis image analysis.

## 4. Results

**Figure 6:**
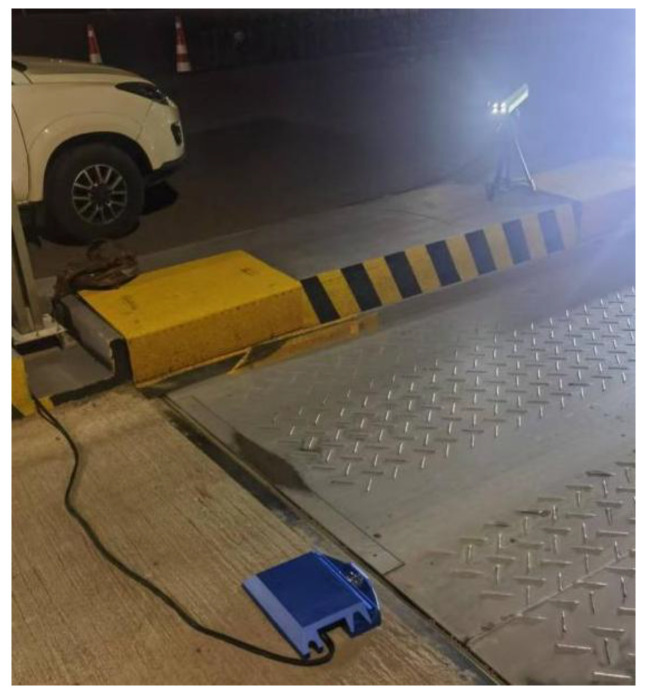
Field Experiment.

To validate the effectiveness of the proposed method, this study conducted experimental evaluations using a proprietary vehicle chassis area-scan image dataset. This dataset was specifically collected by our research team at the entrance of a highway toll station (the on-site setup is shown in Figure 6) to generate panoramic under-vehicle views through image stitching for subsequent object detection.

The data acquisition was conducted from a low-angle, bottom-up perspective, covering both daytime and nighttime scenarios for lighting diversity. When a vehicle enters the data acquisition zone, the device commences capturing area-scan images at a frequency of 30 Hz. The raw data comprises approximately 180,000 area-scan images, covering vehicle types ranging from 2 to 6 axles. Each individual area-scan image has a fixed resolution of 2048 × 160 pixels and captures a lateral segment of the vehicle chassis.

For the continuously captured images, this article employed keyframe extraction and image enhancement preprocessing combined with the ORB feature detection and matching algorithm, and incorporated an FB module for feature enhancement.

### 4.1. FeatureBooster Fine-Tuning for Linear Scan Data

Before performing feature matching on the images, this article first enhanced the data using median filtering, CLAHE, and gamma correction.

To validate the effectiveness of the image enhancement algorithm, the standard ORB algorithm was first evaluated on the original, unprocessed area scan images. Subsequently, to further improve descriptor discrimination for the specific task of vehicle chassis image analysis, the pre-trained FB was fine-tuned using the collected area-scan camera dataset. The original model, pre-trained on natural images, was adapted to the unique characteristics and challenges of vehicle chassis imagery.

Fine-tuning was performed using a comprehensive dataset of approximately 180,000 area-scan images acquired from toll station operations. The data were divided into training, validation, and testing subsets in an 8:1:1 ratio. In the self-boosting stage of FB, the keypoint encoder is configured as a four-layer MLP (with the structure [32, 64, 128, 256]); the descriptor encoder is set as a two-layer MLP (with the structure [256, 512]) for preprocessing, enhancing its feature representation through residual connections. In the cross-boosting stage of FB, the model employs an AFTAttention-based module for contextual modeling, ultimately outputting enhanced descriptors of the specified dimension through a projection layer. For domain-specific adaptation of the pre-trained FB model, the AdamW optimizer was employed with β values of (0.9, 0.999), a weight decay of 0.01, an initial learning rate of 1 × 10^−4^, and a batch size of 8 across 50 epochs. Gradient clipping was implemented with a norm threshold of 1.0 to maintain training stability. A ReduceLROnPlateau scheduler was used to reduce the learning rate by half (factor = 0.5) when the validation loss stagnated, with a lower learning rate bound set at 1 × 10^−7^. Early stopping was introduced with a patience of 10 epochs. To combat overfitting, label smoothing was applied with a smoothing factor of 0.1. The boosting loss coefficient (λ) was fixed at 10, in accordance with the reference implementation.

**Figure 7:**
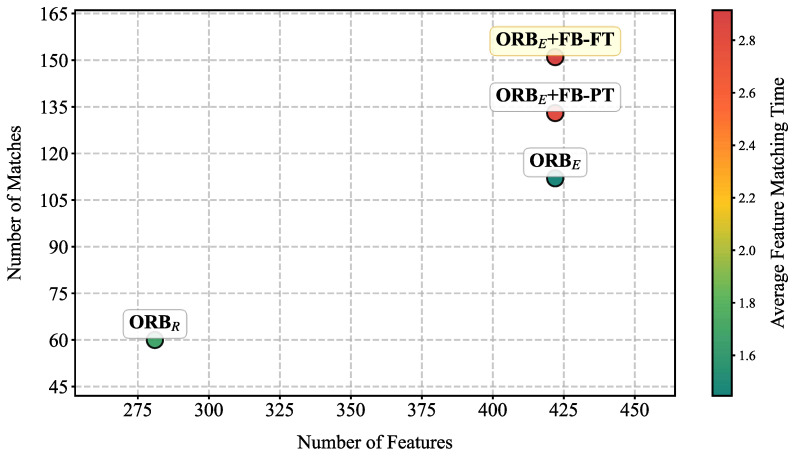
Performance comparison of different ORB models. *X*-axis is the number of Features detected, *Y*-axis is the number of correctly matched features, and the color reflects the average feature matching time of the model.

To quantitatively evaluate the improvement in feature matching brought by the fine-tuned FB, the average number of features, matches and average feature matching time were compared across four configurations: ORB on raw images (ORB_R_), ORB on enhanced images (ORB_E_), ORB_E_ combined with the pre-trained FB model, and ORB_E_ combined with the fine-tuned FB model. The results, summarized in Table 1 and Figure 7, demonstrate a substantial performance gain. The results indicate that image enhancement preprocessing alone provides a boost in ORB’s features (ORB_R_: 281 ± 31, ORB_E_: 422 ± 21). After fine-tuned FB enhancement, an average of 151 ± 18 matches can be obtained, which is higher than the results of ORB_E_ (112 ± 21) and pre-trained FB enhancement (133 ± 18). The baseline ORB_R_ achieved the fastest matching time (5.79 millisecond). This time increased to 7.11 millisecond for ORB_E_, attributable to the higher number of features processed. The incorporation of the FB model introduced additional overhead, resulting in matching times of 10.06 millisecond and 9.97 millisecond for the pre-trained and fine-tuned versions, respectively. Despite this increase, the processing times remain viable for real-time application, given the substantial improvement in matching quality.

**Figure 8:**
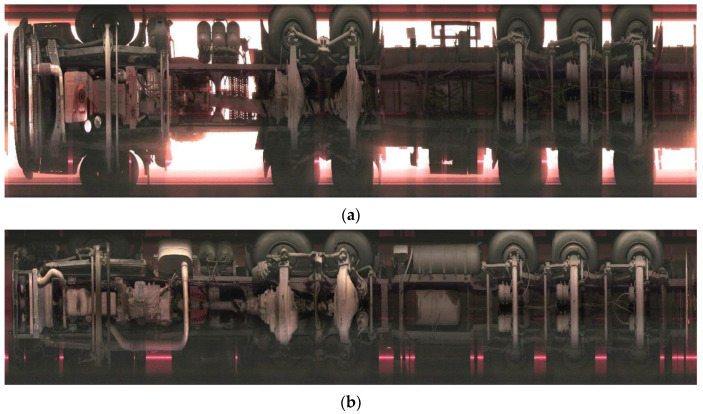
The panoramic image of the vehicle chassis after image stitching. (**a**) Daytime; (**b**) Nighttime.

This approach ultimately achieved high-quality panoramic reconstruction of vehicle chassis images. Typical examples of vehicle chassis reconstruction results are shown in Figure 8a,b correspond to daytime and nighttime scenes).

### 4.2. Model Comparison on ORB Baseline Dataset

The vehicle chassis panoramic image dataset constructed in Section 3.1 was used as the input data for the YOLO algorithm in this study. The dataset includes 2032 vehicles, covering the following axle types: 2-axle vehicles (817), 3-axle vehicles with single drive (173), 3-axle vehicles with double drive (39), 4-axle vehicles with single drive (142), 4-axle vehicles with double drive (61), 5-axle vehicles with single drive (124), 5-axle vehicles with double drive (29), 6-axle vehicles with single drive (154), and 6-axle vehicles with double drive (493). The total number of drive axles is 2654, and the total number of driven axles is 5075. All images were annotated by a single annotator using LabelImg to ensure consistency. The dataset was randomly split into training, validation, and test sets with an 8:1:1 ratio, and then fed into multiple versions of the YOLO object detection algorithm for training and evaluation to investigate the performance of different models in the vehicle chassis detection task.

To ensure a fair comparison across models, consistent hyperparameters were applied during training: an initial learning rate of 0.01 decayed via a cosine annealing scheduler, a batch size of 32, and a total of 300 epochs. Optimization was performed using AdamW with a weight decay of 0.0005 and momentum set to 0.937. Gradient norm clipping was employed at a threshold of 10.0 to enhance stability. All input images were resized to 640 × 640 pixels while preserving their original aspect ratios through letter-box scaling. Data augmentation encompassed Mosaic (disabled in the last 10 epochs), MixUp with α = 0.5, random affine transformations (rotation within ±5°, translation ±0.1, scaling ±0.5), and color jittering in the form of hue (±0.015), saturation (±0.7), and value (±0.4) adjustments. Label smoothing with a factor of 0.1 was incorporated to reduce overfitting.

To comprehensively assess model performance, representative models including YOLOv5s, YOLOv6n, YOLOv7-tiny, YOLOv8n, YOLOv9t, YOLOv10n, and YOLOv11n were selected for comparative experiments. Precision (P), Recall (R), Object detection accuracy (mAP@50, mAP@50:95) and speed (FPS) were used as the key evaluation metrics.

**Figure 9:**
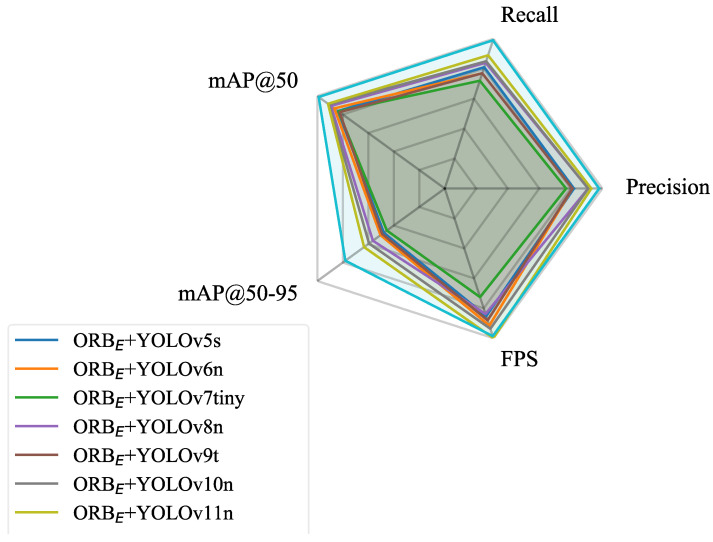
Performance comparison of different YOLO models on vehicle chassis object detection task.

**Figure 10:**
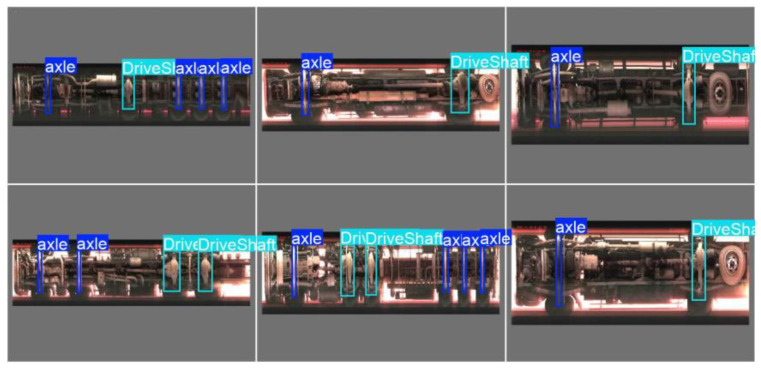
Some of the model’s prediction results.

**Figure 11:**
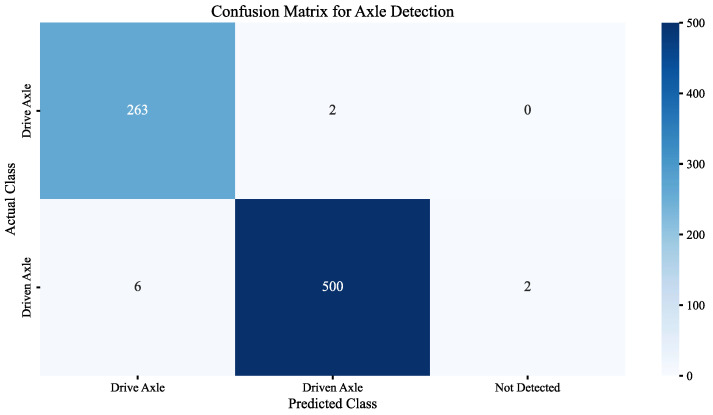
The confusion matrix of ORB + FB + YOLOv11n.

The performance comparison of each model on the test set is detailed in Table 2 and Figure 9, and some of the model’s prediction results are shown in Figure 10. Analysis of the data reveals the following:

- On the dataset constructed from images stitched solely by the ORB algorithm, the detection accuracies (mAP@50 and mAP@50:95) of the various YOLO versions were generally similar, with YOLOv11n achieving the best performance (mAP@50: 0.916 ± 0.012, mAP@50:95: 0.633 ± 0.015). Notably, YOLOv11n also achieved the highest Precision (0.93) and Recall (0.89) among the YOLO-based models, reflecting its enhanced ability to accurately detect target objects while reducing false negatives, which is consistent with its mAP performance.
- Notably, when using images stitched by the ORB_E_+ FB-FT to build the dataset and employing the YOLOv11n model for detection, the performance of the model was improved (mAP@50: 0.989 ± 0.010, mAP@50:95: 0.780 ± 0.012). This performance leap is further underscored by Precision (0.98) and Recall (0.99) achieved by the ORB + FB + YOLOv11n model. These values indicate that FB enhances the results of image feature matching, leading to a clearer and more complete dataset. As a result, the detection model exhibits a reduction in both false positives and false negatives.
- The confusion matrix (Shown as Figure 11) revealed specific error patterns: 2 drive axles were misclassified as driven axles, while 6 driven axles were misclassified as drive axles. Additionally, there were 2 undetected driven axles (FN). Further examination of these errors suggests that geometric distortion and compression in chassis images—caused by high vehicle speeds during image acquisition—are likely the main contributing factors. Elevated vehicle speeds reduce the spatial resolution of the image sequence, resulting in loss of detail and obscuring discriminative features. This compression effect particularly blurs geometric and structural details that differentiate drive axles from driven axles, thereby amplifying confusion between the two classes. Meanwhile, the compressed axle may resemble the vehicle frame, leading to its non-recognition.

**Figure 12:**
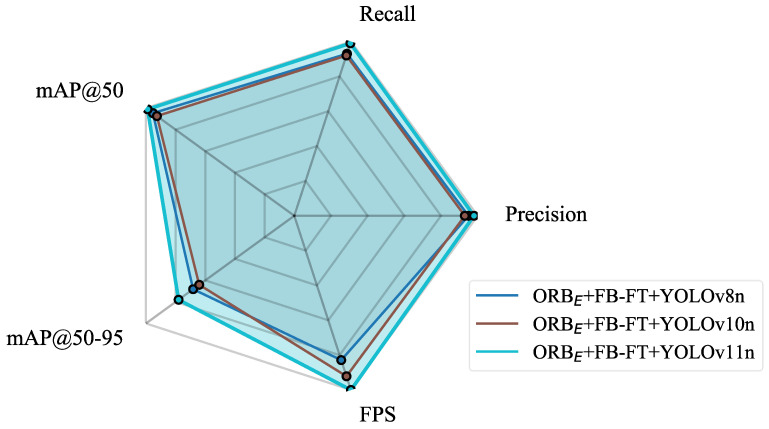
Performance comparison of different YOLO models based on ORB_E_ + FB-FT.

To further evaluate the impact of image stitching quality, an ablation study was conducted using the ORB_E_ + FB-FT with YOLOv8n and YOLOv10n, building upon their baseline performances observed in Table 2. The results in Table 3 show that the improved stitching boosted detection accuracy across both models. While YOLOv8n and YOLOv10n exhibited a gain, YOLOv11n achieved the highest overall performance, see Figure 12.

### 4.3. Robustness Analysis in Nighttime Scenarios

**Figure 13:**
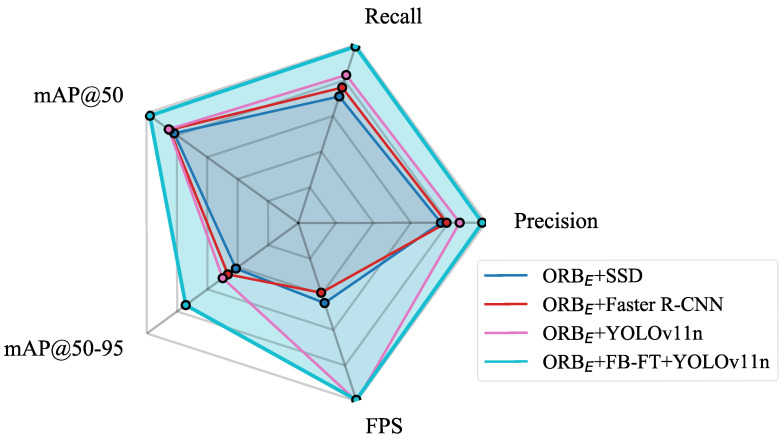
Performance comparison of different models on object detection tasks in nighttime scenarios.

Considering the complexity of lighting conditions in practical application scenarios, especially the challenges posed by low-light environments at night to image quality and feature extraction, this study focused on robustness analysis in nighttime scenarios. A test set based on nighttime data was additionally constructed to specifically evaluate the model’s performance. To verify the effectiveness of the proposed algorithm, comparative experiments were conducted with current mainstream object detection algorithms, including SSD [26], Faster R-CNN [24], and others. The experimental results are presented in Table 4 and Figure 13.

The analysis of the results in Table 2 leads to the following conclusions:

- Under low-light conditions at night, the detection accuracy of the YOLOv11n model based on pure ORB-stitched images decreases (compared to daytime/overall test set, mAP@50 drops from 0.916 to 0.853, a decline of approximately 6.3%; mAP@50:95 decreases from 0.633 to 0.499, a reduction of about 13.4%). This indicates that insufficient lighting will affect the feature extraction and matching quality of the ORB algorithm, resulting in stitched images with more noise, blurring, or mismatched regions, which in turn degrades the performance of subsequent object detection models. However, compared to SSD and Faster R-CNN, YOLOv11n demonstrates relatively superior speed and accuracy, indicating that its performance better meets the requirements for algorithm precision and real-time processing in on-site vehicle chassis image recognition.
- In stark contrast, the YOLOv11n model using ORB + FB stitched images maintains high detection accuracy and stability in nighttime scenarios (P: 0.98, R: 0.99, mAP@50: 0.977 ± 0.011, mAP@50:95: 0.743 ± 0.012). Compared to the pure ORB approach under nighttime conditions, its P and R improved by 0.12 and 0.16, while mAP@50 and mAP@50:95 improved by 12.4% and 24.4%, respectively. This fully demonstrates that the FB module effectively enhances image features, improving the robustness of the ORB algorithm in challenging environments such as low illumination.

### 4.4. On-Site Real-Time Performance Validation

After completing the model training, real-time on-site testing was conducted at the toll station to further verify the model’s performance and check for potential overfitting. During this testing, information from 200 vehicles was continuously collected both during the day and at night. By analyzing the model’s average processing time and accuracy, the system’s precision and real-time performance were validated. Throughout the testing process, the system was able to output the stitched image within 2 s after each vehicle passed, and the axle type recognition accuracy reached 99%. These results indicate that the model does not exhibit overfitting and that the device can effectively perform real-time data acquisition and feature recognition.

## 5. Discussion

**Figure 14:**
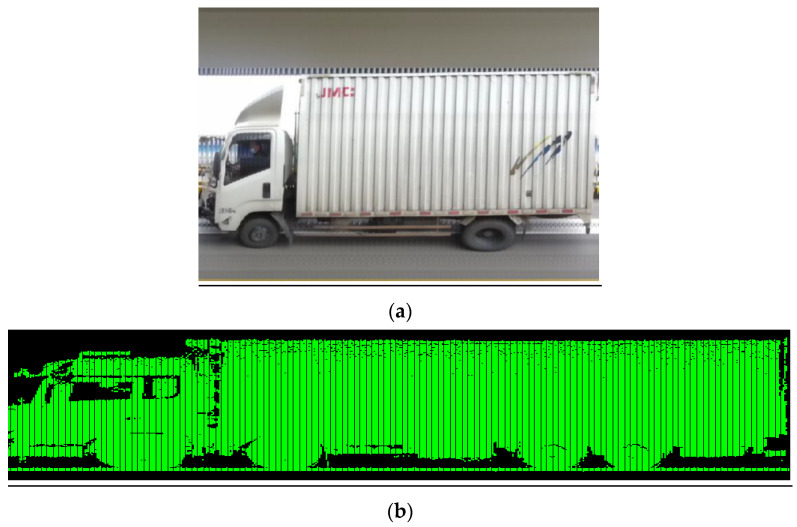
Images output by axle type recognition devices installed roadside. (**a**) Image-based axle recognition device collected data; (**b**) laser-based axle recognition device collected data, with the green section indicating the vehicle contour.

With the progressive implementation of relevant policies in China, research on panoramic vehicle chassis imaging technology has gained increasing significance. On one hand, assisting in identifying axle types can enhance operational efficiency at highway toll stations and help alleviate traffic congestion. On the other hand, chassis image can provide verifiable evidence for toll collection, reducing errors and disputes. This article presents an integrated portable area array-based vehicle image acquisition and axle feature recognition system. Compared to embedding inductive coils or piezoelectric sensors, this device can not only identify the number of axles but also distinguish between drive and driven axles of vehicles, thereby better supporting the axle-based toll collection system at highway toll stations. Moreover, the device can be directly placed on the road surface without the need for road construction, offering flexible deployment and low installation requirements. For non-contact devices such as image-based or radar-based sensors deployed roadside, while they can effectively recognize axle types, the results are often not intuitive or easily distinguishable (as shown in Figure 14). This further demonstrates that the device developed in this study can effectively address this issue.

**Figure 15:**
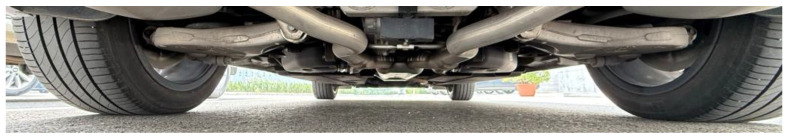
Image acquisition by lateral camera.

However, due to limitations in the device’s field of view, effective tire and lifted axle identification remains challenging. At the current stage, the device has undergone upgrades to enable this functionality by incorporating a lateral camera (as shown in Figure 15), and comprehensive testing and validation have been conducted. The results will be presented in detail in a future document.

**Figure 16:**
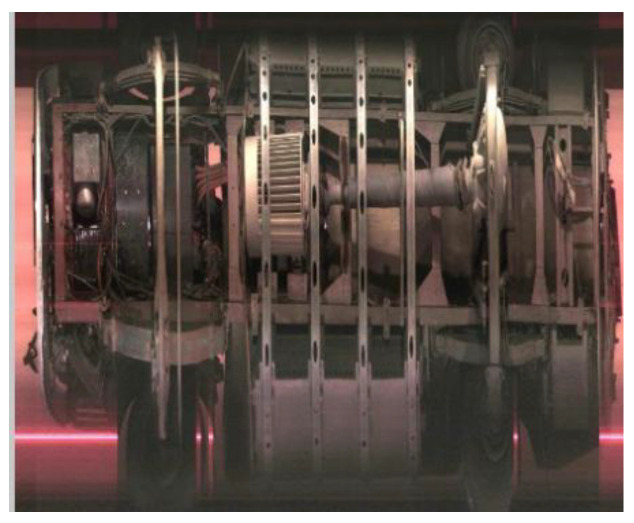
Axle image distortion caused by excessive vehicle speed.

In the context of image feature matching, this study incorporates keyframe selection to alleviate the problem of data redundancy resulting from fluctuations in vehicle speed. This strategy helps decrease computational overhead and storage requirements while improving matching efficiency. Nevertheless, at high vehicle speeds, localized distortions become apparent in the stitched chassis panoramas, as illustrated in Figure 16. When such distortion occurs near axle regions, it compresses the corresponding image areas and adversely affects subsequent feature extraction. To overcome this challenge, subsequent work will explore the integration of a velocity sensing module within the acquisition system for real-time speed monitoring. The measured speed data will be input to the keyframe sampling and stitching algorithms to dynamically adjust the acquisition rate or introduce motion-aware compensation. This enhancement is expected to improve the system’s responsiveness to speed changes, mitigating image compression and geometric distortion.

The ORB algorithm features fast computation speed, making it highly compatible with real-time stitching of vehicle chassis images. However, ORB may exhibit a relatively high mismatch rate under complex environments, repetitive image textures, and rapid object movements [47]. In this study, an FB module was incorporated to enhance image features, improving the accuracy of feature matching, which is clearly reflected in subsequent image detection tasks.

This study validates the effectiveness of the proposed portable imaging system and the integrated stitching-and-detection algorithm through panoramic vehicle chassis imagery. The promising experimental results confirm the feasibility of the overall approach under controlled conditions. However, to facilitate practical deployment, more comprehensive evaluations are necessary. Future work will include a detailed cost–benefit analysis, large-scale statistical validation across multiple toll stations, and rigorous testing under diverse real-world scenarios—such as rain, snow, varying lighting, and complex traffic flows. Additionally, it is crucial to conduct robustness analyses under these diverse and complex traffic scenarios to ensure the reliability and adaptability of the system in various conditions. These evaluations will further assess the system’s robustness and generalization capabilities.

## 6. Conclusions

This study proposes and implements a real-time vehicle chassis panoramic image acquisition and processing system based on area-scan cameras and image processing technologies. The equipment can be deployed to the site within 20 min without embedding. By integrating keyframe extraction, ORB feature detection and matching, and FB feature enhancement algorithms, the system successfully addresses the challenge of poor image stitching quality under complex lighting conditions, especially in low-light nighttime environments. Field tests demonstrate that the system can stably and efficiently identify the number of vehicle axles, providing a reliable technical foundation for subsequent scene detection.

In terms of vehicle chassis target recognition, this research constructs datasets based on different image stitching algorithms and systematically evaluates the performance of various YOLO series object detection models. Experimental results indicate:

- **Enhanced feature matching through fine-tuning:** After domain-specific fine-tuning of the FB on area scan data, the system achieved an average of 151 ± 18 feature matches, outperforming both the pre-trained FB enhancement (133 ± 18) and the baseline ORB on enhanced images (ORB_E_: 112 ± 21). This fine-tuning process further optimized the model’s adaptability to vehicle chassis imagery, contributing to more stable and accurate stitching results.
- **Feature enhancement:** Incorporating the FB module into the ORB algorithm substantially improves the accuracy (overall P increased to 0.98, R increased to 0.99, mAP@50 increased to 0.989 ± 0.010, mAP@50:95 increased to 0.780 ± 0.012) and robustness (nighttime scene P maintained at 0.98, R at 0.99,mAP@50 at 0.977 ± 0.011, mAP@50:95 at 0.743 ± 0.012) of the subsequent YOLOv11n model in vehicle chassis target detection tasks.
- **Effectiveness of algorithm combination:** The proposed ORB_E_ + FB-FT + YOLOv11n scheme effectively overcomes the instability of traditional ORB feature extraction under low-light conditions, achieving high-quality image stitching and high-precision, real-time axle recognition of vehicle chassis images in complex field environments.
- In summary, the main contributions of this study are as follows:

A real-time system for acquiring high-quality panoramic images of vehicle chassis was developed, with the core innovation being the application of the FB module to enhance the robustness of ORB feature extraction. Extensive experiments validated the effectiveness of the proposed ORB + FB image stitching scheme and demonstrated that, when combined with the advanced YOLOv11n object detection model, it can provide technical support for the acquisition of vehicle chassis images and feature detection in field environments. Through further detailed field validation and equipment optimization, this technology can be widely applied in areas such as highway overload control, toll station removal, vehicle identity verification, and chassis inspection of new energy vehicles.

## Figures and Tables

**Table 1:** Performance comparison of different ORB models.

Models	Features	Matches	Average Feature Matching Time (Millisecond)
ORB_R_	281 ± 31	60 ± 37	5.79
ORB_E_	422 ± 21	112 ± 21	7.11
ORB_E_ + FB-PT (Pre-trained)	422 ± 21	133 ± 18	10.06
ORB_E_ + FB-FT (Fine-tuned)	422 ± 21	151 ± 20	9.97

**Table 2:** Performance comparison of different YOLO models on vehicle chassis object detection task.

Models	P	R	MAP50	MAP50-95	FPS
ORB_E_ + YOLOv5s	0.82	0.81	0.837 ± 0.015	0.478 ± 0.018	121
ORB_E_ + YOLOv6n	0.81	0.77	0.866 ± 0.013	0.504 ± 0.016	130
ORB_E_ + YOLOv7tiny	0.77	0.72	0.834 ± 0.016	0.454 ± 0.019	103
ORB_E_ + YOLOv8n	0.91	0.84	0.893 ± 0.012	0.564 ± 0.014	119
ORB_E_ + YOLOv9t	0.81	0.77	0.825 ± 0.017	0.491 ± 0.018	125
ORB_E_ + YOLOv10n	0.91	0.85	0.908 ± 0.011	0.594 ± 0.013	133
ORB_E_ + YOLOv11n	0.93	0.89	0.916 ± 0.012	0.633 ± 0.015	142
ORB_E_ + FB-FT +YOLOv11n	0.98	0.99	0.989 ± 0.010	0.780 ± 0.012	140

**Table 3:** Performance comparison of different YOLO models based on ORB_E_ + FB-FT.

Models	P	R	MAP50	MAP50-95	FPS
ORB_E_ + FB-FT + YOLOv8n	0.95	0.96	0.953 ± 0.014	0.682 ± 0.015	116
ORB_E_ + FB-FT + YOLOv10n	0.93	0.92	0.926 ± 0.015	0.641 ± 0.017	129
ORB_E_ + FB-FT + YOLOv11n	0.98	0.99	0.989 ± 0.010	0.780 ± 0.012	140

**Table 4:** Performance comparison of different models on object detection tasks in nighttime scenarios.

Models	P	R	MAP50	MAP50-95	FPS
ORB_E_ + SSD	0.76	0.71	0.817 ± 0.018	0.412 ± 0.021	62
ORB_E_ + Faster R-CNN	0.79	0.76	0.848 ± 0.014	0.465 ± 0.018	54
ORB_E_ + YOLOv11n	0.86	0.83	0.853 ± 0.017	0499 ± 0.019	138
ORB_E_ + FB-FT + YOLOv11n	0.98	0.99	0.977 ± 0.011	0.743 ± 0.012	137

## Data Availability

The data are not publicly available due to privacy. The data are available on reasonable request from the corresponding author, subject to approval from our industrial partner.

## Appendix A

**Table A1:** The identification of overloaded and overweight freight vehicles on highways.

Total Number of Axles	Vehicle Model	Illustration	Axle Type Example	Driven Axle Count	Drive Axle Count	Weight Limit (Tons)
2-Axle	Goods Vehicle			1	1	18
3-Axle	Centre-axle Trailer Combination			2	1	27
	Articulated Vehicle			2	1	
	Goods Vehicle			1	2	25
				2	1	
4-Axle	Centre-axle Trailer Combination			3	1	36
				2	2	35
	Articulated Vehicle			3	1	36
	Full Trailer Truck			3	1	
	Goods Vehicle			2	2	31
5-Axle	Centre-axle Trailer Combination			3	2	43
				4	1	
	Articulated Vehicle			3	2	
				4	1	
				4	1	42
	Full Trailer Train			3	2	43
				4	1	
6-Axle	Centre-axle Trailer Combination			4	2	49
				5	1	46
				4	2	49
				5	1	46
	Articulated Vehicle			4	2	49
				5	1	46
				5	1	46
	Full Trailer Train			4	2	49
				5	1	46
Remarks	The total weight of a two-axle goods vehicle and its cargo shall not exceed the gross vehicle weight specified on the vehicle registration certificate. Except for the drive axle, for the two-axle and three-axle groups as well as semi-trailers and full trailers shown in the illustration, the total mass limit shall be reduced by 3 tons for every two tires removed. For trailers equipped with tires having a nominal section width of no less than 425 mm and the vehicle combinations they form, as well as goods vehicles equipped with drive axles fitted with tires of nominal section width no less than 445 mm and their vehicle combinations, the total mass limit shall not be reduced. When the drive axle is equipped with dual tires on each side and air suspension, the total mass limit for three-axle and four-axle goods vehicles shall each increase by 1 ton; for four-axle articulated vehicle combinations where the drive axle is equipped with dual tires on each side and air suspension, and the distance between the two axles of the semi-trailer d ≥ 1800 mm, the total mass limit shall be 37 tons. For vehicle types not listed in the illustration, the corresponding total mass limits shall be determined according to the “Limits of dimensions, axle load and masses for motor vehicles, trailers and combination vehicles” (GB 1589-2016). For violations of vehicle and cargo dimension limits, implementation shall proceed in phased steps according to unified deployment by relevant national authorities. Before such deployment, no inspections of vehicle dimensions shall be conducted. Vehicles transporting hazardous chemicals that illegally exceed weight or dimension limits shall be punished by public security authorities in accordance with Article 88 of the “Regulations on the Safety Management of Hazardous Chemicals.” Vehicles transporting fresh agricultural products that illegally exceed weight or dimension limits shall not be granted toll exemptions when using toll roads; when using non-toll roads, the main approach shall be criticism and education without immediate penalties. Remediation efforts for trailer combinations carrying standard containers shall be separately deployed. Before such special remediation, the focus shall be on inspecting whether the total vehicle and cargo weight exceeds the load limit standard, with no inspections of vehicle dimensions conducted temporarily. Remediation efforts for low-bed semi-trailers transporting general cargo shall be separately deployed. Before such special remediation, the focus shall be on addressing violations of total vehicle and cargo weight exceeding load limits and the use of fake or duplicate license plates.					
